# An evolutionarily diverged CCD4 enzyme negatively regulates mesocotyl elongation in rice

**DOI:** 10.1111/nph.70799

**Published:** 2025-12-02

**Authors:** Yasha Zhang, Abdugaffor Ablazov, Aparna Balakrishna, Chakravarthy Rajan, Yagiz Alagoz, Kit Xi Liew, Lamis Berqdar, Jian You Wang, Ikram Blilou, Xiongjie Zheng, Salim Al‐Babili

**Affiliations:** ^1^ Center of Excellence for Sustainable Food Security King Abdullah University of Science and Technology (KAUST) Thuwal 23955‐6900 Saudi Arabia; ^2^ The Plant Science Program, Biological and Environmental Science and Engineering Division King Abdullah University of Science and Technology Thuwal 23955‐6900 Saudi Arabia; ^3^ The Laboratory of Plant Cell and Developmental Biology (LPCDB), Biological and Environmental Science and Engineering Division King Abdullah University of Science and Technology Thuwal 23955‐6900 Saudi Arabia; ^4^ The BioActives Lab, Biological and Environmental Science and Engineering Division King Abdullah University of Science and Technology Thuwal 23955‐6900 Saudi Arabia; ^5^ Agricultural Biotechnology Research Center Academia Sinica 115 Taipei Taiwan; ^6^ Biotechnology Center in Southern Taiwan, Academia Sinica 74145 Tainan Taiwan; ^7^ National Key Laboratory for Germplasm Innovation & Utilization of Horticultural Crops Huazhong Agricultural University Wuhan 430070 China; ^8^ Hubei Hongshan Laboratory Wuhan 430070 China

**Keywords:** 3‐OH‐β‐cyclocitral, abscisic acid, apocarotenoids, carotenoid cleavage dioxygenase, mesocotyl*Oryza sativa*

## Disclaimer

The New Phytologist Foundation remains neutral with regard to jurisdictional claims in maps and in any institutional affiliations.

## Introduction

Carotenoids constitute a large class of natural isoprenoid compounds synthesized in all photosynthetic organisms and by many nonphotosynthetic microorganisms (Moise *et al*., 2014; Rodriguez‐Concepcion *et al*., 2018; Li *et al*., 2025). In plants, carotenoids contribute to the pigmentation of fruits and flowers, thereby promoting pollination and seed dispersal by attracting animals and insects (Yuan *et al*., 2015). More critically, they play vital roles by safeguarding the photosynthetic apparatus from photooxidative damage and participate in light harvesting, thereby enabling plant growth and survival (Hashimoto *et al*., 2016).

Due to their conjugated double‐bond structures, carotenoids are highly susceptible to oxidative cleavage, producing a wide array of biologically active metabolites known as apocarotenoids. These include the precursors of the phytohormones such as strigolactones (SLs) and abs*cis*ic acid (ABA), and growth regulators such as anchorene, β‐cyclocitral, zaxinone, as well as diverse pigments and volatile compounds (Schwartz *et al*., 1997; Wang *et al*., 2019; Moreno *et al*., 2021; Li *et al*., 2025). The biosynthesis of apocarotenoids is catalyzed by the *carotenoid cleavage dioxygenases* (*CCD*) gene family, which comprises several subfamilies: 9‐*cis*‐epoxycarotenoid dioxygenases (NCEDs), CCD1, CCD4, CCD7, CCD8, CCD2 (identified in *Crocus* species), and Zaxinone Synthase (ZAS) (Walter & Strack, 2011; Frusciante *et al*., 2014; Ahrazem *et al*., 2016; Jia *et al*., 2018; Wang *et al*., 2019; Ablazov *et al*., 2023a). Each CCD subfamily exhibits distinct substrate specificities and cleavage site preferences, resulting in the formation of diverse apocarotenoids with particular biological functions (Zheng *et al*., 2020). NCED enzymes catalyze the cleavage of the C9‐C10 double bond in 9′‐*cis*‐neoxanthin and/or 9‐*cis*‐violaxanthin to produce the ABA precursor xanthoxin (Schwartz *et al*., 1997). CCD7 and CCD8 sequentially convert 9‐*cis*‐β‐carotene into carlactone, a central intermediate in strigolactone biosynthesis (Alder *et al*., 2012; Al‐Babili & Bouwmeester, 2015; J. Y. Wang *et al*., 2024). ZAS converts hydroxy‐apocarotenoids, primarily 3‐OH‐β‐apo‐10′‐carotenal, into zaxinone, a growth regulator identified in rice and other plants (Wang *et al*., 2019; Ablazov *et al*., 2020). CCD1 enzymes catalyze the cleavage of multiple carotenoids and apocarotenoid substrates at different double bonds, yielding a wide range of volatile compounds and dialdehydes (Simkin *et al*., 2004; IIg *et al*., 2014). CCD2L cleaves zeaxanthin to yield crocetin dialdehyde, the precursor of crocin, the pigment responsible for saffron coloration (Frusciante *et al*., 2014; Ahrazem *et al*., 2016).

CCD4 enzymes exhibit remarkable diversity in substrate specificity and regio‐selectivity across plant species, reflecting their broad functional divergence (Zheng *et al*., 2020). For instance, CCD4 enzymes from *Arabidopsis thaliana* and *Solanum tuberosum* cleave bicyclic carotenoids at the C9′‐C10′ and/or C9–C10 double bonds, producing C_13_ volatiles and C_27_ apocarotenoids that are supposed to be further degraded to colorless compounds (Gonzalez‐Jorge *et al*., 2013; Bruno *et al*., 2016; Mi & Al‐Babili, 2019). By contrast, *Citrus* CCD4b cleaves carotenoids, particularly zeaxanthin, at the C7–C8 or C7′–C8′ positions, yielding the C_30_ apocarotenoid β‐citraurin (also known as 3‐OH‐β‐apo‐8′‐carotenal), which contributes to the characteristic red coloration of citrus fruit peel (Ma *et al*., 2013; Rodrigo *et al*., 2013; Zheng *et al*., 2019, 2021). Similarly, BdCCD4.1/3 and GjCCD4a from *Buddleja davidii* and *Gardenia jasminoides*, respectively, cleave carotenoids at both C7–C8 and C7′–C8′ sites, forming crocetin dialdehyde, the precursor of the saffron pigment crocin (Ahrazem *et al*., 2017; Xu *et al*., 2020; Zheng *et al*., 2022; Lobato‐Gómez *et al*., 2025). In *Crocus*, CCD4 enzymes cleave carotenoids at the C9–C10 or C9′–C10′ position to generate C_13_ volatiles (Rubio *et al*., 2008; Rubio‐Moraga *et al*., 2014). However, knowledge of CCD4 enzymes in monocot species remains limited. In particular, it is unclear whether CCD4 enzymes from cereals differ in substrate specificity and region‐selectivity from their dicot homologs.

The annotation of *LOC*_*Os12g24800*, a rice gene encoding a putative carotenoid cleavage enzyme, has varied across studies. While some have classified it as *CCD4b* (Yang *et al*., 2017; Ko *et al*., 2018; Choi *et al*., 2025), others have referred to it as *NCED2* (Oliver *et al*., 2007; Lyu *et al*., 2013; Mao *et al*., 2017; Chen *et al*., 2021; Gao *et al*., 2022; Jin *et al*., 2023a, 2023b; J. D. Wang *et al*., 2024), suggesting a role in ABA biosynthesis. This divergence in annotation likely arises from sequence similarity within conserved regions and the lack of functional validation at the enzymatic level. Given the distinct cleavage specificities and resulting physiological functions of *CCD4* and *NCED* subfamilies (Schwartz *et al*., 1997; Zheng *et al*., 2020), resolving the biochemical identity of *LOC*_*Os12g24800* is essential for accurately interpreting its biological function. Several previous studies, guided by the NCED2 annotation, have examined its expression dynamics and promoter regulation in ABA‐related responses (Oliver *et al*., 2007; Lyu *et al*., 2013; Mao *et al*., 2017; Chen *et al*., 2021; Gao *et al*., 2022; Jin *et al*., 2023a, 2023b; Wang *et al*., 2024b). However, without biochemical validation, it remains unclear whether these observations accurately reflect the true enzymatic function of the encoded protein. To clarify its enzymatic identity and physiological role, we performed a comprehensive analysis integrating phylogenetic analysis, *in vitro* and *in planta* enzymatic assays, and phenotypes of corresponding mutants. Our findings demonstrate that *LOC*_*Os12g24800* encodes a CCD4‐type enzyme with cleavage specificity distinct from NCEDs, clarifying that it does not participate directly in ABA biosynthesis and resolving previous ambiguities arising from its NCED2 annotation. Thus, this study provides a biochemical and functional framework that helps reconcile previous interpretations and guides future research on carotenoid cleavage and apocarotenoid signaling in rice.

## Materials and Methods

### Plant materials and phenotyping

The transgenic callus of *Citrus paradise* Macf. overexpressing *tpCrtB* and *OsBCH* (Zheng *et al*., 2022) was grown in the dark at room temperature and subcultured at 20‐d intervals. *Nicotiana benthamiana* plants were grown in a growth chamber at 24°C, under a 12 h day‐night photoperiod. Rice seedlings were grown in a Biochamber at a day/night temperature of 27/25°C, under a 12‐h day/night photoperiod. The *d14‐1* and *d3‐1* mutants (cv. Shiokari) were described previously (Ishikawa *et al*., 2005). For mesocotyle phenotyping, we have either used vermiculite or ½‐strength Murashige & Skoog medium (1/2MS) medium consisting of 0.5% (w/v) agar. The vermiculite experiment was performed as described by Patil *et al*. (2019) with some modifications. Initially, 250 ml of vermiculite was added to the glass vessels (6 cm diameter, 18 cm height). Then, *c*. 20 (dry) seeds were placed on this layer, and following this, 500 ml of vermiculite was added on top of the seeds. Last, 400 ml of Milli‐Q water (pH 5.8) was added to each bottle, and each was tightly closed and incubated in complete darkness under the above conditions for 10 d. Mesocotyl lengths were scanned and measured from the seminal root emergence point to the coleoptile node using ImageJ software. For agar mesocotyl phenotyping, unshelled rice seeds were sterilized with 1.5% sodium hypochlorite for 30 min and then germinated at 30°C for 1 d. Uniform seeds were selected and placed on solid 1/2MS medium consisting of 0.5% (w/v) agar medium with the indicated concentrations of chemicals (3‐OH‐β‐cyclocitral and acetone as mock) and then grown in the dark at 30°C for 7 d. The mesocotyl length measurement was conducted manually or via ImageJ. For seedling phenotyping, 1‐wk‐old seedlings were grown hydroponically in Hoagland nutrient solution (Wang *et al*., 2019) for 3 wk, and phenotypic data were recorded. The solution was changed every other day.

Mesocotyl Embedding, Sectioning, and Staining: Mesocotyl samples were first vacuum‐infiltrated in 4% (w/v) paraformaldehyde prepared in 1 × PBS buffer (pH 6.9) at room temperature for 1–2 h. After fixation, samples were embedded in 5% (w/v) low‐melting‐point agarose, and longitudinal sections (80‐μm‐thick) were obtained using a Leica VT1200S vibratome. To visualize cell walls, sections were stained overnight with 0.1% (v/v) SR2200, following the protocol described by Musielak *et al*. (2016). Confocal images were acquired using a Zeiss LSM 710 inverted microscope with a 405 nm excitation laser, and fluorescence signals were collected in the 430–500 nm emission range. Vertical sections within the coleoptile node to the base of the seminal root were analyzed. Cell lengths within the mesocotyl region were measured using ImageJ software.

### In bacterial and *vitro* assays

The coding sequences of *OsCCD4b* (LOC_Os12g24800) and *OsCCD4a* (LOC_Os02g47510), excluding the chloroplast transit peptide region, were cloned into the pThio vector in frame with an N‐terminal thioredoxin tag. Primers used for plasmid construction are listed in Table S1. Constructs pThio‐AtNCED2 and pThio‐CitCCD4b were obtained from previous studies (Zheng *et al*., 2021; Jia *et al*., 2022). pThio‐OsCCD4b, pThio‐OsCCD4a, pThio‐CitCCD4b, and the void plasmid were transformed into *E. coli* competent cells engineered to accumulate either β‐carotene or zeaxanthin. In bacterial assays, UHPLC analysis of enzymatic products was conducted as described previously (Zheng *et al*., 2022).

For *in vitro* assay, the pThio‐OsCCD4b, pThio‐OsCCD4b, and pThio‐AtNCED2 were transformed into BL21 *E. coli* harboring the pGro7 plasmid. *E. coli* cell induction, crude lysates and substrates preparation, incubation conditions, metabolites extraction, and UHPLC analysis were conducted as previously described (Bruno *et al*., 2016; Zheng *et al*., 2022).

Carotenoids and apocarotenoids were analyzed using an UHPLC‐DAD system and a C_30_ YMC Carotenoid column (150 × 3 mm, 5 μm). The mobile phases consisted of (A) Methanol/MTBE in a 1 : 1 ratio and (B) Methanol/MTBE/Water in a 30 : 1 : 10 ratio. Chromatographic separation was carried out at a flow rate of 0.6 ml min^−1^. For *in bacteria* samples, the elution program began with 100% solvent B, linearly decreasing to 0% over 15 min, and held until 24 min. For *in vitro* enzymatic products, the gradient transitioned from 100% B to 45% B within 15 min, stepped to 0% B over the next 5 min, and maintained this condition until 24 min. Subsequently, the gradient returned to 100% B within 1 min and held until 33 min.

### Plasmid construction and Transient expression in *N. benthamiana* leaves

The CDS of *OsCCD4b* (LOC_Os12g24800) was cloned into the pDONR221 vector to generate pDONR221‐OsCCD4b, respectively, by using ‘BP’ reaction kit (Invitrogen). pDONR221‐OsCCD4b was then recombined into the pB2GW7 overexpression vector using an ‘LR’ reaction kit (Invitrogen) to generate pB2GW7‐OsCCD4b. The primers used for plasmid construction are listed in Table S1.

The constructs pB2GW7‐OsCCD4b and pB2GW7‐AtCCD4 (At4g19170) from our previous study (Zheng *et al*., 2021), along with the empty vector (EV), were individually electroporated into *Agrobacterium tumefaciens* strain GV3101. The Agrobacterium‐mediated transient expression was performed by using leaves of 5‐wk‐old *N. benthamiana* plants as previously described (Zheng *et al*., 2022). The pB2‐OsCCD4b and EV agrobacterium cell pellets were resuspended in infiltration buffer to an OD_600_ of 0.5; the OD_600_ of the p19 agrobacterium cell culture was 0.3. The pB2‐OsCCD4b‐transformed agrobacteria were mixed with p19 agrobacterial culture in a 1 : 1 ratio. The agro‐infiltrated leaves were harvested after 5 d, then frozen in liquid nitrogen for the following apocarotenoid profiling.

### Supertransformation of engineered callus

The abovementioned pB2GW7‐OsCCD4b and pB2GW7‐AtCCD4 were electroporated into *A. tumefaciens* EHA105, respectively. The 20‐d‐old transgenic callus was incubated with suspension (OD = 0.3) EHA105 harboring the above‐constructed pB2GW7‐OsCCD4b or pB2GW7‐AtCCD4 in liquid MS B5 medium for 10 min. After 3 d of co‐cultivation, the transformed callus was selected in MS B5 solid medium with 50 mg l^−1^ glufosinate‐ammonium (Sigma‐Aldrich) for 5 wks. The solid and liquid MS B5 medium used in this study was according to Zheng *et al*. (2023). The small piece of each recovered transformed callus was then transferred onto selective solid medium and sub‐cultured for at least six cycles. Twenty‐day‐old transgenic lines grown in solid medium without antibiotics were harvested for later apocarotenoid profiling and molecular analysis.

### Generation of *OsCCD4b* overexpression and CRISPR mutant lines in rice

The aforementioned pDONR221‐OsCCD4b was recombined into the pH7WG2D overexpression vector using an ‘LR’ reaction kit (Invitrogen) to generate pH 7‐OsCCD4b. The plasmid was electroporated into Agrobacterium tumefaciens (EHA105) and was transformed into Nipponbare wild‐type *Japonica* rice cultivar as previously described (Hiei & Komari, 2008). The transgenic rice seedlings were selected by 50 mg l^−1^ hygromycin in a 1/2 MS medium. Twelve seedlings from each T1 transgenic line were grown to identify homozygote lines at the T2 generation. Transgenic lines' seeds that showed a 90–100% germination rate on a 1/2 MS medium containing 50 mg l^−1^ hygromycin were considered homozygote lines and then were propagated to obtain T3 generation seeds. The *OsCCD4b* mutant in the Nipponbare background using the clustered regularly interspaced short palindromic repeat (CRISPR)‐Cas9 technology was previously generated (Yang *et al*., 2017). The *OsCCD4b* mutant in Zhonghua 11 (*O. sativa* L. *japonica*, ZH11) background was generated by Biogle (Lu *et al*., 2017) using the CRISPR–Cas9‐mediated editing method. The transgenicity and mutagenicity of the CRISPR mutant lines were confirmed as described by Ablazov *et al*. (2023b).

### Quantitative Real‐time PCR

Total RNA was isolated using the Trizol method as previously described (Zheng *et al*., 2023). First‐strand cDNA synthesis was performed with the iScript™ cDNA synthesis Kit (Bio‐Rad) following the manufacturer's protocol. Quantitative real‐time polymerase chain reaction was conducted on a CFX384 Touch RT‐PCR System (Bio‐RAD) using SsoAdvanced™ Universal SYBR® Green Supermix (Bio‐Rad) Kit. Relative gene expression levels were calculated using the E^−ΔΔCt^ method. The qRT‐P2CR primers were provided in Table S1.

### UHPLC‐HR‐MS analysis of apocarotenoids

Apocarotenoids were extracted from freeze‐dried tissues according to a previous protocol (Mi *et al*., 2018). Dried extracts were re‐dissolved in 90 : 10 (v/v) acetonitrile/water, followed by filtration through a 0.22 μm filter, and subjected to LC‐MS analysis. The separation and detection of all apocarotenoids except 3‐OH‐β‐cyclocitral were conducted using UHPLC coupled with a Q‐Orbitrap high‐resolution mass spectrometer (HR‐MS), as previously described (Mi *et al*., 2018; Zheng *et al*., 2022). Detection of 3‐OH‐β‐cyclocitral followed a separate UHPLC‐HR‐MS method optimized for glycosylated apocarotenoids (Mi *et al*., 2019). Apocarotenoids were identified and quantified by comparing retention times and MS spectra with authentic and isotope‐labeled internal standards (Buchem B.V., Apeldoorn, the Netherlands) as previously described (Mi *et al*., 2018).

### ABA quantification using UHPLC–MS

For ABA analysis, *c*. 5 mg freeze‐dried powder was extracted twice with 600 μl of 10% methanol containing 1% acetic acid and internal standard (D6‐ABA, 1 ng each sample) in an ultrasound bath for 5 min, followed by incubation on ice for 30 min. After centrifugation, the two extracts were combined and filtered with a 0.22 μm filter before UHPLC–MS analysis. Identification and quantitative analysis of ABA were performed as previously described (Jia *et al*., 2022).

### UHPLC analysis of carotenoids and chlorophylls

Approximately 10–20 mg of freeze‐dried tissue powder was extracted on ice for 20 min using a mixture of chloroform and methanol (2 : 1, v/v), as described by Fraser *et al*. (2000). After adding 1 volume of water, the mixture was centrifuged to allow phase separation. The lower organic phase was collected, vacuum‐dried, and used for UHPLC analysis of carotenoids and chlorophylls. UHPLC separation and detection were performed as previously described in Zheng *et al*. (2023), using mobile phase A (Methanol/Methyl tert‐butyl ether, 50 : 50, v/v) and mobile phase B (Methanol/water/methyl tert‐butyl ether, 6 : 3 : 1, v/v/v) at a flow rate of 0.8 ml/min and a column temperature of 35°C. The gradient started at 30% A/70% B, linearly increased to 100% A over 19 min, held until 34 min, then returned to initial conditions by 36 min, and maintained until 38 min. Compounds were identified by comparing retention times and absorption spectra with authentic standards (CaroteNature, Switzerland) and published data (Fraser *et al*., 2007). Quantification was performed using standard calibration curves.

### Phylogenetic tree and conserved motif analysis

The phylogenetic tree was constructed using the Neighbor‐Joining method in MEGA5 software (Tamura *et al*., 2011), with 1000 bootstrap replicates. Protein accession numbers used for phylogenetic analysis are listed in the legend of Fig. S1. The conserved motifs and specific residues within the CCD sequences of both rice and Arabidopsis were examined through the MEME web server (http://meme‐suite.org/tools/meme) (Bailey *et al*., 2009). The server settings were configured with a minimum conserved motif width of 15 and a maximum of 40, allowing for up to 10 motifs per theme. All other parameters were set to their default values.

### RNA‐seq experiment and bioinformatic analysis

Total RNA was extracted from rice mesocotyls of the *OsCCD4b* CRISPR mutant and the overexpression lines using TRI‐Reagent with Direct‐zol RNA MiniPrep Kit according to the manufacturer's instructions (Zymo Research, Irvine, CA, USA). Seedlings that were 9 d old, with 4–5 seedlings grouped together for each sample, were collected in a dark room illuminated by green lights. The quality and quantity of RNA were assessed using the NanoDrop 6000. Library preparation, sequencing, and subsequent data analysis were conducted by Novogene Technology. Sequencing took place on the Illumina Novaseq platform, yielding 150 bp paired‐end reads. The clean paired‐end reads were aligned to the Nipponbare reference genome (http://rice.uga.edu/pub/data/Eukaryotic_Projects/o_sativa/annotation_dbs/pseudomolecules/version_7.0/) utilizing Hisat2 v.2.0.5. To identify differentially expressed genes, the DESeq. 2R package (v.1.20.0) was employed under the criteria of an adjusted *P*‐value ≤ 0.05 and a fold change (FC) ≥ 0.58. PCA was conducted to illustrate consistency among biological replicates. The clusterProfiler R package was used for GO enrichment analysis of the DEGs through the NovoMagic online platform (https://ap‐magic.novogene.com/), considering GO terms with a corrected *P*‐value ≤0.05 as significantly enriched by the differentially expressed genes.

### Statistical analysis

All the data are presented as mean ± SEM from at least three biological replicates unless otherwise noted. Statistical significance was assessed using GraphPad Prism v8 and Microsoft Excel 2010. Significant differences are denoted as *P‐value <0.05, **P < 0.01, and ***P < 0.001.

## Results and Discussion

**Fig. 1:**
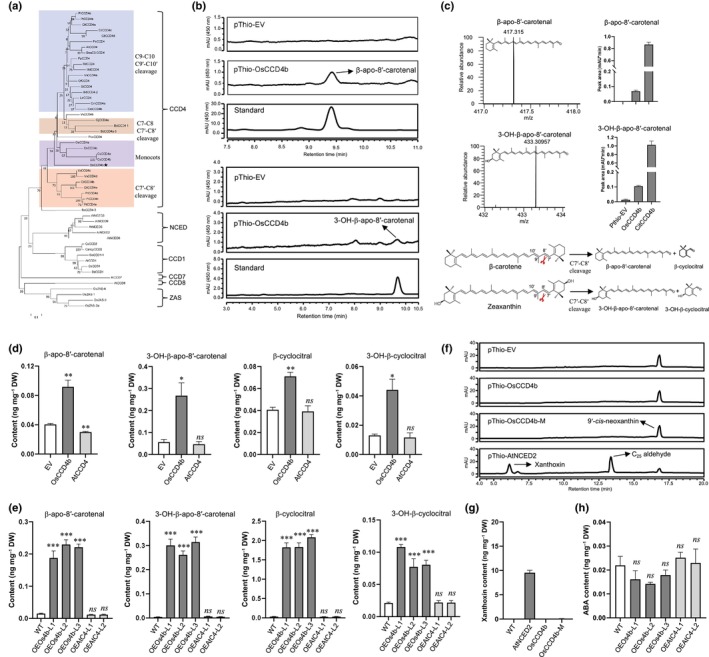
Enzymatic activity of OsCCD4b *in vivo* and *in vitro*. (a) Phylogenetic analysis of CCD proteins from different species. Accession numbers were obtained from previous studies (Wang *et al*., 2019; Zheng *et al*., 2022). *LOC_Os12g24800* encoded protein, referred to as OsCCD4b, clusters within the CCD4 subfamily and is closely related to monocot CCD4 enzymes, which form a distinct subgroup within the CCD4 family. The CCD4 proteins are separated into four groups, consistent with their cleavage site specificity. (b) UHPLC analysis of OsCCD4b *in vivo* enzymatic activity using β‐carotene and zeaxanthin as substrates. OsCCD4b catalyzed the formation of β‐apo‐8′‐carotenal and 3‐OH‐β‐apo‐8′‐carotenal from β‐carotene and zeaxanthin, respectively. (c) Relative quantification of apocarotenoid products from *in vivo* assays. The cleavage products were confirmed by UHPLC‐HR‐MS. (d) Relative quantification of apocarotenoids in *Nicotiana* leaves transiently expressing *OsCCD4b* or *AtCCD4*. (e) Relative quantification of apocarotenoids in stable transgenic callus lines overexpressing *OsCCD4b* (*OEOs4b*) or *AtCCD4* (OEAtC4). (f) UHPLC analysis of *in vitro* cleavage activity of OsCCD4b, its mutant variant OsCCD4b‐M, and AtNCED2 using 9′‐*cis*‐neoxanthin as a substrate. Only *AtNCED2* cleaved the C11′–C12′ double bond to produce xanthoxin and a C_25_ aldehyde. (g) Quantification of xanthoxin in *in vitro* enzymatic reaction products (h). Relative quantification of abscisic acid (ABA) in stable transgenic callus lines overexpressing *OsCCD4b* (*OEOs4b*) or *AtCCD4* (OEAtC4). Identification and quantification of apocarotenoids and ABA were performed using UHPLC‐HR‐MS. Bars represent SEM. Asterisks indicate statistically significant differences by Student's *t*‐test: *, *P* < 0.05; **, *P* < 0.01; ***, *P* < 0.001. *ns* indicates no significant difference. EV, empty vector; DW, dry weight; mAU, milli‐absorbance units.

We first constructed a phylogenetic tree of CCD proteins from diverse plant species, revealing that *LOC*_*Os12g24800* encoded protein clusters within the CCD4 subfamily clade (Fig. 1a). In parallel, motif analysis of rice and Arabidopsis CCD proteins revealed notable differences in motif composition among subfamilies: NCEDs, CCD4s, CCD1s, ZASs, CCD8s, and CCD7s contained 15, 13, 11, 11, 7, and 7 conserved motifs, respectively (Supporting Information Fig. S1). We found that motifs 14 and 15, characteristic of NCED family members, such as *AtNCED2/3/5/9* and *OsNCED3/4/5*, are absent in LOC_Os12g24800 and CCD4 proteins (Fig. S1). These findings support the classification of LOC_Os12g24800, which we call in the following CCD4b, as a CCD4‐type enzyme rather than a member of the NCED subfamily. Furthermore, its phylogenetic separation from Arabidopsis CCD4 indicated that it may possess distinct enzymatic activity.

To investigate the enzymatic activity of OsCCD4b, we expressed the gene in *Escherichia coli* strains engineered to accumulate either β‐carotene or zeaxanthin. UHPLC (Ultra‐High‐Performance Liquid Chromatography) analysis of cell extracts revealed the production of β‐apo‐8′‐carotenal and 3‐OH‐β‐apo‐8′‐carotenal, respectively, but not β‐apo‐10′‐carotenal or 3‐OH‐β‐apo‐10′‐carotenal (Fig. 1b,c). LC‐MS‐based apocarotenoid profiling confirmed the UHPLC results (Fig. S2), indicating that OsCCD4b preferentially cleaves the C7′–C8′ double bond to generate C_30_ and C_10_ apocarotenoids. This cleavage specificity differs from that of Arabidopsis CCD4, which targets the C9–C10 or C9′–C10 double bond, but resembles the activity of the CitCCD4b, consistent with their phylogenetic clustering (Fig. 1a). We also investigated the second rice CCD4 enzyme encoded by *LOC*_*Os02g47510*, referred to here as OsCCD4a, using the same *in vivo* assay system. However, we did not detect cleavage of β‐carotene under our experimental conditions (Fig. S2).

Given the relatively low enzymatic activity of OsCCD4b in the bacterial system, we further evaluated its function *in planta*. Transient expression of *OsCCD4b* in *Nicotiana benthamiana* leaves significantly increased the levels of β‐apo‐8′‐carotenal and 3‐OH‐β‐apo‐8′‐carotenal, as well as their corresponding C_10_ co‐products, β‐cyclocitral and 3‐OH‐β‐cyclocitral (Fig. 1d), but not of C9′–C10′ cleavage products (Fig. S3), further supporting the cleavage specificity inferred from bacterial assays.

To corroborate these findings, we generated stably transformed citrus callus lines overexpressing *OsCCD4b* in a background preengineered to accumulate carotenoids via the introduction of bacterial *PSY* (*phytoene synthase*) and *BCH* (*β‐carotene hydroxylase*) genes (Fig. S4). UHPLC‐Q‐Orbitrap‐MS analysis revealed a significant increase in β‐apo‐8'‐carotenal and 3‐OH‐β‐apo‐8'‐carotenal, along with their respective co‐products, β‐cyclocitral and 3‐OH‐β‐cyclocitral (Fig. 1e). By contrast, callus overexpressing *AtCCD4* did not show an increase in C_30_ apocarotenoid levels. Likewise, no increase in C9′–C10′ cleavage products was detected in *OsCCD4b*‐overexpressing citrus callus (Fig. S5), consistent with the results obtained from both *Nicotiana* leaves and *in bacterio* assays. We also observed that overexpression of *OsCCD4b* resulted in a significant reduction in total carotenoid content, including β‐carotene and zeaxanthin (Fig. S6). In light of the previous annotation of *LOC*_*Os12g24800* encoded protein as NCED2, we conducted *in vitro* enzymatic assays with 9'‐*cis*‐neoxanthin as a substrate and the Arabidopsis NCED2 (AtNCED2) as a positive control. As expected, AtNCED2 efficiently converted 9'‐*cis*‐neoxanthin into the ABA precursor xanthoxin (Figs 1f,g, S7). By contrast, we did not detect xanthoxin in the OsCCD4b incubation, which supports its distinct enzymatic activity. Confirming the results of the *in vitro* assay, ABA levels remained unchanged in *OsCCD4b*‐overexpression citrus callus lines (Fig. 1h). We also tested *OsCCD4bM*, a mutant variant previously suggested to enhance ABA content *in planta* (Huang *et al*., 2024); however, we did not observe any formation of xanthoxin (Fig. 1f,g). Confirming the stereo‐specificity and excluding the direct contribution to ABA biosynthesis, OsCCD4b did not convert 9‐*cis*‐β‐carotene (Fig. S8). Overall, our enzymatic and transgenic analyses demonstrate that OsCCD4b preferentially cleaves at the C7'–C8' double bond to produce C_30_ and C_10_ apocarotenoids, highlighting an evolutionary divergence in cleavage‐site specificity among CCD4 enzymes. While CCD4s in dicots such as *A. thaliana* and *S. tuberosum* predominantly catalyze the cleavage of the C9–C10 or C9′–C10 double bonds to generate C_13_ volatiles, monocot CCD4s show distinct regio‐selectivity. Notably, while its cleavage activity closely resembles that of Citrus CCD4, the two genes differ in expression patterns, suggesting functional divergence. While *Citrus CCD4* is predominantly expressed in the fruit peel (Rodrigo *et al*., 2013; Zheng *et al*., 2019), *OsCCD4b* shows only weak expression in rice vegetative tissues. Moreover, the catalytic activity of OsCCD4b is markedly lower than that of its citrus counterpart (Fig. 1c), suggesting a limited role in bulk carotenoid degradation in rice. Given that certain apocarotenoids function as signaling molecules at low concentrations, OsCCD4b may instead contribute to the regulation of plant growth and development through the production of specific bioactive cleavage products.

**Fig. 2:**
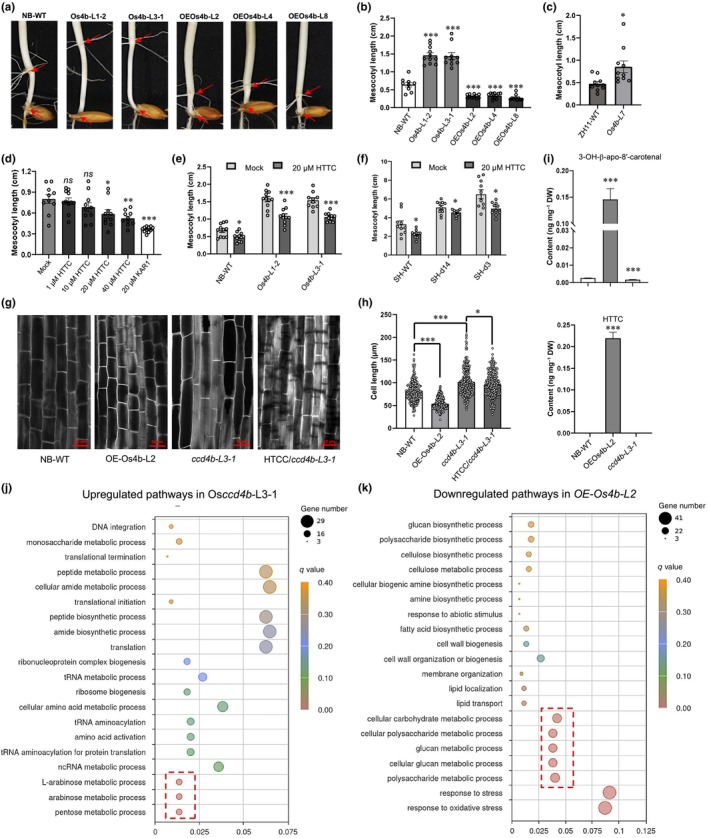
*OsCCD4b* regulates mesocotyl length and cell elongation through its cleavage product 3‐OH‐β‐cyclocitral. (a) Mesocotyls of seedlings grown in darkness for 9 d. The upper and lower arrows indicate the positions of the coleoptile node and the basal part of the seminal root, respectively. The mesocotyl is the tissue between these two arrows. (b) Mesocotyl length of *OsCCD4b* overexpression lines and CRISPR knockout mutants in the NB (Nipponbare) background. (c) Mesocotyl length of *OsCCD4b* CRISPR mutants in the ZH11 (Zhonghua 11) background. (d) Effects of different concentrations of HTTC (3‐OH‐β‐cyclocitral) on mesocotyl length. KAR1, a known negative regulator of mesocotyl elongation, was used for comparison. (e) Effects of 20 μM HTTC treatment on mesocotyl elongation in 8‐d‐old dark‐grown Nipponbare WT and *Osccd4b* CRISPR mutant seedlings. (f) Effects of 20 μM HTTC treatment on mesocotyl elongation in 8‐d‐old dark‐grown *d14* and *d3* mutants in the SH (Shiokari) background. (g) Representative tissue sections of mesocotyls from the wild‐type (WT), *OsCCD4b* overexpressing line, *Osccd4b* CRISPR knockout mutant, and mutant treated with HTTC. Red bars represent 50 μm. (h) Cell length measurements were performed using ImageJ. (i) Quantification of 3‐OH‐β‐apo‐8′‐carotenal and HTTC levels in *OsCCD4b* overexpression and CRISPR mutant lines. (j) Gene Ontology (GO) term analysis of upregulated genes in mesocotyl of *Osccd4b* knockout mutants from RNA‐seq. (k) GO term analysis of downregulated genes in mesocotyl of *OsCCD4b* overexpression lines from RNA‐seq. GO terms with corrected *P‐*value ≤ 0.05 were considered significantly enriched by up‐ or downregulated genes. Bars represent SEM. Asterisks indicate statistically significant differences by Student's *t*‐test: *, *P* < 0.05; **, *P* < 0.01; ***, *P* < 0.001. *ns* indicates no significant difference. DW, dry weight.

To further investigate its potential biological function, we examined the *OsCCD4* (*LOC*_*Os12g24800*) locus and observed that it colocalizes with quantitative trait loci and genome‐wide association study signals associated with mesocotyl length (Liu *et al*., 2019; Zhan *et al*., 2020). However, its direct functional relevance to this trait has not been experimentally validated. Therefore, we first screened previously reported *Osccd4b* CRISPR lines (Yang *et al*., 2017) and identified a Cas9‐free mutant (Figs S9, S10), which exhibited significantly elongated mesocotyls (Fig. 2a,b). To confirm this phenotype, we generated an independent *OsCCD4b* knockout line in the ZH11 background (Fig. S11), which similarly displayed longer mesocotyls (Fig. 2c). Confirming the role of the enzyme as a negative regulator of the mesocotyl length, overexpression of *OsCCD4b* in the Nipponbare background (Fig. S12) led to shorter mesocotyls (Fig. 2a,b). Apart from the mesocotyl phenotype, we did not detect significant differences in other traits, including root growth on solid media (Fig. S13) as well as root length, shoot length, shoot biomass, or root biomass in hydroponically grown seedlings (Fig. S14).

Since CCD enzymes exert their function through the production of apocarotenoids, we hypothesized that a downstream metabolite of OsCCD4b may mediate its effect on mesocotyl elongation. We focused on 3‐OH‐β‐cyclocitral, one of the main products of OsCCD4b cleavage, as β‐cyclocitral is highly volatile and difficult to apply consistently. Exogenous application of 3‐OH‐β‐cyclocitral significantly suppressed mesocotyl elongation in a dose‐dependent manner, with concentrations above 20 μM causing significant inhibitory effects (Fig. 2d). However, its activity was weaker than that of the karrikin KAR1 (3‐methyl‐2H‐furo[2,3‐c]pyran‐2‐one), a known growth‐regulating compound that negatively affects mesocotyl length. Notably, 3‐OH‐β‐cyclocitral treatment partially rescued the elongated mesocotyl phenotype of the *OsCCD4b* CRISPR knock‐out mutant (Fig. 2e), suggesting that this apocarotenoid may function downstream of OsCCD4b. Given that carotenoids are also precursors for SLs, we tested whether 3‐OH‐β‐cyclocitral acts through known SL or karrikin (KAR) signaling pathways. However, SL‐ and KAR‐insensitive mutant responded similarly to wild‐type plants upon 3‐OH‐β‐cyclocitral treatment (Fig. 2f), indicating that its mode of action is likely independent of these hormone signaling pathways.

To understand the underlying cellular mechanism, we measured the cell length of the mesocotyl in the dark‐grown seedlings. The *OsCCD4b* CRISPR knockout line exhibited increased cell length, whereas overexpression lines showed shorter cells. Moreover, exogenous application of 3‐OH‐β‐cyclocitral partially rescued the elongated cell phenotype in the mutant line (Fig. 2g,h). Consistent with these observations, apocarotenoid profiling revealed increased levels of 3‐OH‐β‐cyclocitral and its co‐product of 3‐OH‐β‐apo‐8′‐carotenal in both shoots and roots of *OsCCD4b*‐overexpressing plants, and reduced levels in the CRISPR knock‐out mutants (Figs 2i, S15, S16).

To determine whether 3‐OH‐β‐cyclocitral is derived from zeaxanthin, we quantified carotenoids in both *OsCCD4b* knockout and overexpression lines. Overexpression of *OsCCD4b* led to a significant decrease in β‐carotene and zeaxanthin, whereas other carotenoids remained largely unchanged. Interestingly, the CRISPR knockout mutant did not show a significant difference in total carotenoid levels (Fig. S17), suggesting that OsCCD4b is not a major regulator of overall carotenoid content or tissue pigmentation in rice, in contrast to CCD4 enzymes in some other species (Gonzalez‐Jorge *et al*., 2013; Ma *et al*., 2013; Zheng *et al*., 2021). Chl content and ABA level were also unaffected (Figs S18, S19), further supporting the conclusion that OsCCD4b is not directly involved in ABA biosynthesis. This conclusion is consistent with the findings mentioned above, which show the enzyme's inability to generate the ABA precursor xanthoxin (Fig. 1f) and its functional independence from SL and KAR signaling pathways (Fig. 2f), suggesting that 3‐OH‐β‐cyclocitral may act as an independent apocarotenoid signal.

To investigate how OsCCD4b influences mesocotyl and cell development at the transcriptomic level, we performed RNA‐sequencing on mesocotyl tissues from the WT, *Osccd4b* knockout mutant, and overexpression lines. PCA revealed clear transcriptomic separation among the three genotypes (Fig. S20), suggesting that *OsCCD4b* exerts a substantial impact on gene expression in rice mesocotyls. GO (Gene Ontology) enrichment analysis of differentially expressed genes indicated significant alterations in sugar‐related metabolic pathways. Specifically, the *OsCCD4b* knock‐out mutant showed upregulation of genes involved in pentose and arabinose metabolism (Fig. 2j), whereas the overexpression lines displayed downregulation of genes related to polysaccharide and glucan metabolism (Fig. 2k), indicating that *OsCCD4b* may regulate cell elongation, at least in part, by modulating sugar metabolism.

## Conclusion

In conclusion, this study highlights the functional diversification of CCD4 enzymes and the evolutionary emergence of distinct C_30_ and C_10_ apocarotenoids across plant species. Our findings reveal the biological function of the *LOC*_*Os12g24800* gene as *OsCCD4b*, regulating mesocotyl elongation and sugar metabolism through a previously uncharacterized, hormone‐independent apocarotenoid signaling pathway. This work advances our understanding of apocarotenoid biology – particularly the function of 3‐OH‐β‐cyclocitral – in monocot species and provides a foundation for future investigations into growth‐regulating carotenoid‐derived metabolites.

## Competing interests

None declared.

## Author contributions

SA‐B conceived the project and supervised the experiments; XZ and SA‐B designed the experiments; YZ, XZ performed callus transformation and q‐RT‐PCR analysis; CR generated rice overexpression lines; YZ and IB performed confocal imaging of mesocotyl cells; YZ, AA, YA, LB, and JYW performed phenotyping; AA, XZ, and YA conducted and analyzed RNA‐seq experiments; XZ and AA carried out bioinformatics analysis; XZ, KXL, and JM performed metabolite analysis. XZ and AB performed transient expression in *Nicotiana*, *in vivo* and *in vitro* assays. YZ, XZ, and AA prepared figures and wrote the manuscript. SA‐B edited and approved the manuscript. YZ and AA contributed equally to this work.

## Supporting information


**Fig. S1** Analysis of conserved motifs in the CCDs protein sequences of Arabidopsis and rice was conducted using MEME software.
**Fig. S2** Quantification of β‐apo‐8′‐carotenal, β‐apo‐10′‐carotenal, and 3‐OH‐β‐apo‐10′‐carotenal in *in vivo* assays of CCD4 using β‐carotene or zeaxanthin as substrates.
**Fig. S3** Apocarotenoid profiling of *Nicotiana* leaves transiently overexpressing *OsCCD4b* or *AtCCD4*.
**Fig. S4** Relative expression levels of transgenes in independent *OsCCD4b‐* or *AtCCD4‐*overexpression citrus callus lines.
**Fig. S5** Apocarotenoid profiling in transgenic citrus callus overexpressing *OsCCD4b* or *AtCCD4*.
**Fig. S6** Carotenoid analysis of wild‐type and *OsCCD4b* or *AtCCD4* overexpressing citrus callus lines.
**Fig. S7** High‐resolution MS and UV/vis spectra of xanthoxin.
**Fig. S8** UHPLC analysis of the *in vitro* assays of OsCCD4b and its mutant using 9‐*cis*‐β‐carotene as substrate.
**Fig. S9** Mutations in *OsCCD4b* CRISPR knockout Nipponbare plants result in truncated proteins with loss of enzymatic activity.
**Fig. S10** Identification of Cas9‐free *OsCCD4b* CRISPR knockout mutant plants.
**Fig. S11** Sequencing analysis confirmed a *OsCCD4b* knockout CRISPR line in the Zhonghua 11 (ZH11) background.
**Fig. S12** qRT‐PCR confirmation of transgene overexpression in *OsCCD4b*‐overexpressing Nipponbare plants.
**Fig. S13** Root phenotypes of wild‐type, *OsCCD4b* CRISPR knock‐out, and overexpression line seedlings grown on agar medium.
**Fig. S14** Phenotypic characterization of hydroponically grown Nipponbare wild‐type and *OsCCD4b* CRISPR knockout mutant seedlings.
**Fig. S15** Quantification of β‐apo‐8′‐carotenal and 3‐OH‐β‐apo‐8′‐carotenal in different *OsCCD4b* overexpression lines and CRISPR knockout mutants.
**Fig. S16** Apocarotenoid profiling in hydroponically grown shoots of Nipponbare wild‐type and *OsCCD4b* overexpression lines.
**Fig. S17** Carotenoid analysis in hydroponically grown shoots of wild‐type, OsCCD4b overexpression lines, and CRISPR knockout mutants.
**Fig. S18** Chlorophyll levels in hydroponically grown shoots of wild‐type, *OsCCD4b* overexpression lines, and CRISPR knockout mutants.
**Fig. S19** ABA quantification in shoots and roots of Nipponbare wild‐type and *OsCCD4b* CRISPR knockout Nipponbare plants.
**Fig. S20** The PCA based on FPKM value of wild‐type (NB), *OsCCD4b* CRISPR knockout mutant, and overexpression lines obtained from RNAseq.
**Table S1** Sequences of primers used in this study.
Please note: Wiley is not responsible for the content or functionality of any Supporting Information supplied by the authors. Any queries (other than missing material) should be directed to the *New Phytologist* Central Office.

## Data Availability

The materials utilized in this research are available through a Material Transfer Agreement (MTA) with the King Abdullah University of Science and Technology (KAUST). The RNA‐sequencing data produced in this study were deposited in ArrayExpress (https://www.ebi.ac.uk/biostudies/arrayexpress) with accession E‐MTAB‐16215. All data and materials supporting this study are provided in the article and in Figs S1–S20 and Table S1.
