# Supplementary material for: An evolutionarily diverged CCD4 enzyme negatively regulates mesocotyl elongation in rice
Source: New Phytol. 2025 Dec 2;249(4):1569–79. doi: 10.1111/nph.70799 (PMC12825410; doi:10.1111/nph.70799)
Supplement: Supplementary file 1 — Fig. S1 Analysis of conserved motifs in the CCDs protein sequences of Arabidopsis and rice was conducted using MEME software. Fig. S2 Quantification of β‐apo‐8′‐carotenal, β‐apo‐10′‐carotenal, and 3‐OH‐β‐apo‐10′‐carotenal in in vivo assays of CCD4 using β‐carotene or zeaxanthin as substrates. Fig. S3 Apocarotenoid profiling of Nicotiana leaves transiently overexpressing OsCCD4b or AtCCD4. Fig. S4 Relative expression levels of transgenes in independent OsCCD4b‐ or AtCCD4‐overexpression citrus callus lines. Fig. S5 Apocarotenoid profiling in transgenic citrus callus overexpressing OsCCD4b or AtCCD4. Fig. S6 Carotenoid analysis of wild‐type and OsCCD4b or AtCCD4 overexpressing citrus callus lines. Fig. S7 High‐resolution MS and UV/vis spectra of xanthoxin. Fig. S8 UHPLC analysis of the in vitro assays of OsCCD4b and its mutant using 9‐cis‐β‐carotene as substrate. Fig. S9 Mutations in OsCCD4b CRISPR knockout Nipponbare plants result in truncated proteins with loss of enzymatic activity. Fig. S10 Identification of Cas9‐free OsCCD4b CRISPR knockout mutant plants. Fig. S11 Sequencing analysis confirmed a OsCCD4b knockout CRISPR line in the Zhonghua 11 (ZH11) background. Fig. S12 qRT‐PCR confirmation of transgene overexpression in OsCCD4b‐overexpressing Nipponbare plants. Fig. S13 Root phenotypes of wild‐type, OsCCD4b CRISPR knock‐out, and overexpression line seedlings grown on agar medium. Fig. S14 Phenotypic characterization of hydroponically grown Nipponbare wild‐type and OsCCD4b CRISPR knockout mutant seedlings. Fig. S15 Quantification of β‐apo‐8′‐carotenal and 3‐OH‐β‐apo‐8′‐carotenal in different OsCCD4b overexpression lines and CRISPR knockout mutants. Fig. S16 Apocarotenoid profiling in hydroponically grown shoots of Nipponbare wild‐type and OsCCD4b overexpression lines. Fig. S17 Carotenoid analysis in hydroponically grown shoots of wild‐type, OsCCD4b overexpression lines, and CRISPR knockout mutants. Fig. S18 Chlorophyll levels in hydroponically grown shoots of wild [file NPH-249-1569-s001.pdf]

Supporting information

New Phytologist Supporting Information

Article title: An Evolutionarily Diverged CCD4 Enzyme Negatively Regulates Mesocotyl Elongation in Rice

Authors: Yasha Zhang†, Abdugaffor Ablazov†, Aparna Balakrishna, Chakravarthy Rajan, Yagiz Alagoz, Kit Xi Liew, Lamis Berqdar, Jian You Wang, Ikram Blilou, Xiongjie Zheng* and Salim Al-Babili *

Article acceptance date: 31 October 2025

Supplementary Figures:

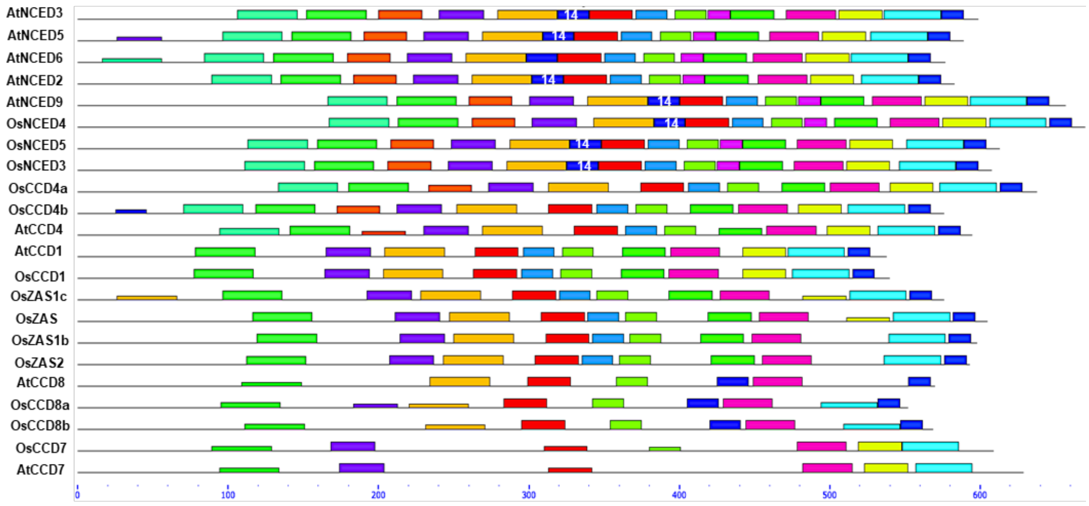

Motif #	Motif sequnces
Motif1	QPTMIHDFAITENYVVIPDQQLVFKLKEM
Motif2	GGGEEDDGYILSFVHDEETGTSELVVIDAKSMELEPVA
Motif3	EIRWVEVPDFFCFHLWNAWEE
Motif4	KLGVGVANTGLVYFAGRLLALSEDDLPYEV
Motif5	TLGRYDFDGQLKSSMIAHPKVDPVTGELFALSYBVVKPPY
Motif6	YIRNGANPLFEPVAGHHLFDGDGMVHAVRIENGSASYYSR
Motif7	KLPSRVPYGFHGTFI
Motif8	SAVALDFPVINPAYLGRKNRYAYAAVADPPPKF
Motif9	QERALGRPVFPKAIGELHGHSGIARLMLF
Motif10	FAKVDLGTGEVKKFDYGEGRFGGEPFFVP
Motif11	DSIFNESDENLRSVLSEIRLNLKTGESTR
Motif12	LERPHPLPKTADPAVQIAGNFAPVGEQPPVRNLPVSGRIP
Motif13	GGSPVVYDKEKVSRFGVLPKY
Motif14	LKYFRFSPDGTKSPDVEIPLD
Motif15	ETDEVVVIGSCMTPP

Figure S1. Analysis of conserved motifs in the CCDs protein sequences of *Arabidopsis* and rice was conducted using MEME software. The parameters set included a minimum motif width of 15 and a maximum of 40, with the capacity to identify as many as 10 motifs for each theme.

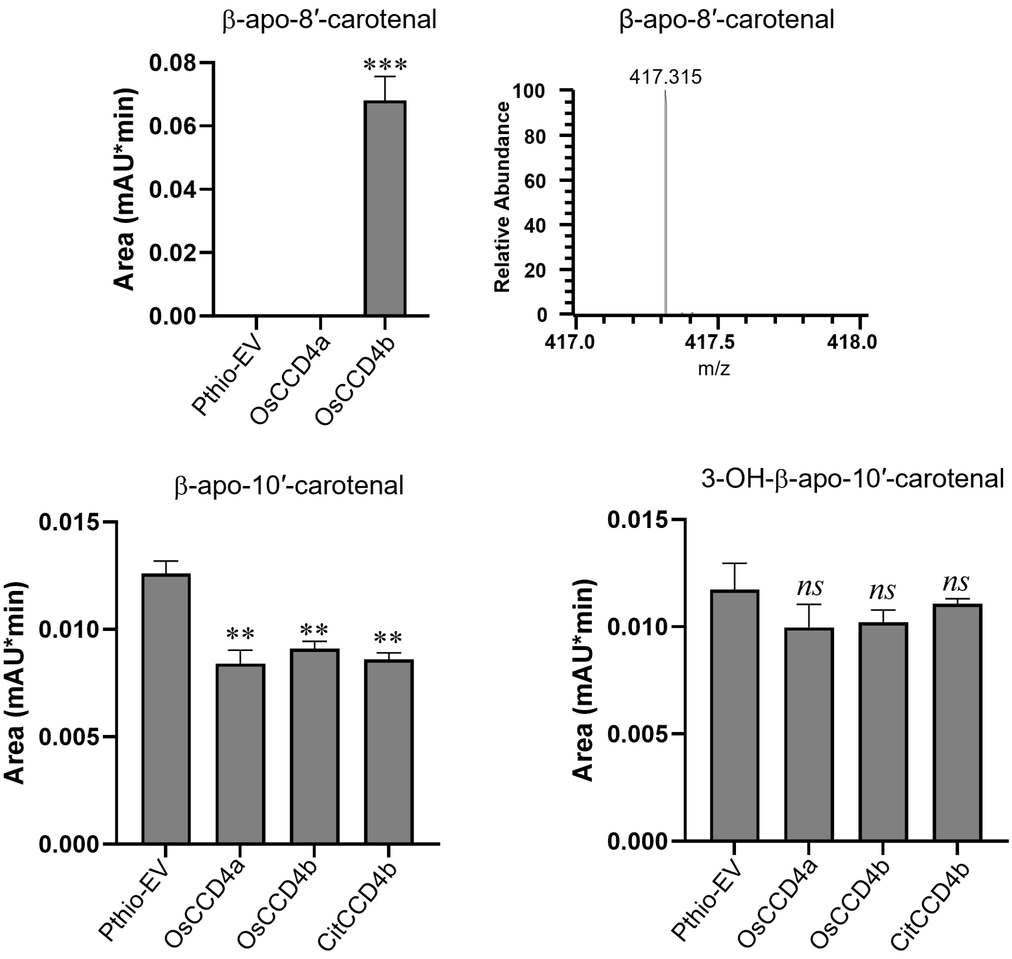

Figure S2. Quantification of β-apo-8′-carotenal, β-apo-10′-carotenal, and 3-OH-β-apo-10′-carotenal in *in vivo* assays of CCD4 using β-carotene or zeaxanthin as substrates. OsCCD4b, but not OsCCD4a, cleaves the C7′–C8′ double bond of β-carotene to produce β-apo-8′-carotenal. Neither OsCCD4b, OsCCD4a, nor citrus CCD4b generate C9′–C10′ cleavage products (β-apo-10′-carotenal and 3-OH-β-apo-10′-carotenal) from β-carotene or zeaxanthin. Bars represent Standard Error of the Mean (SEM). Asterisks indicate statistically significant differences by Student's *t*-test: $^{**}P < 0.01$ and $^{***}P < 0.001$, respectively. "*ns*" indicates no significant difference.

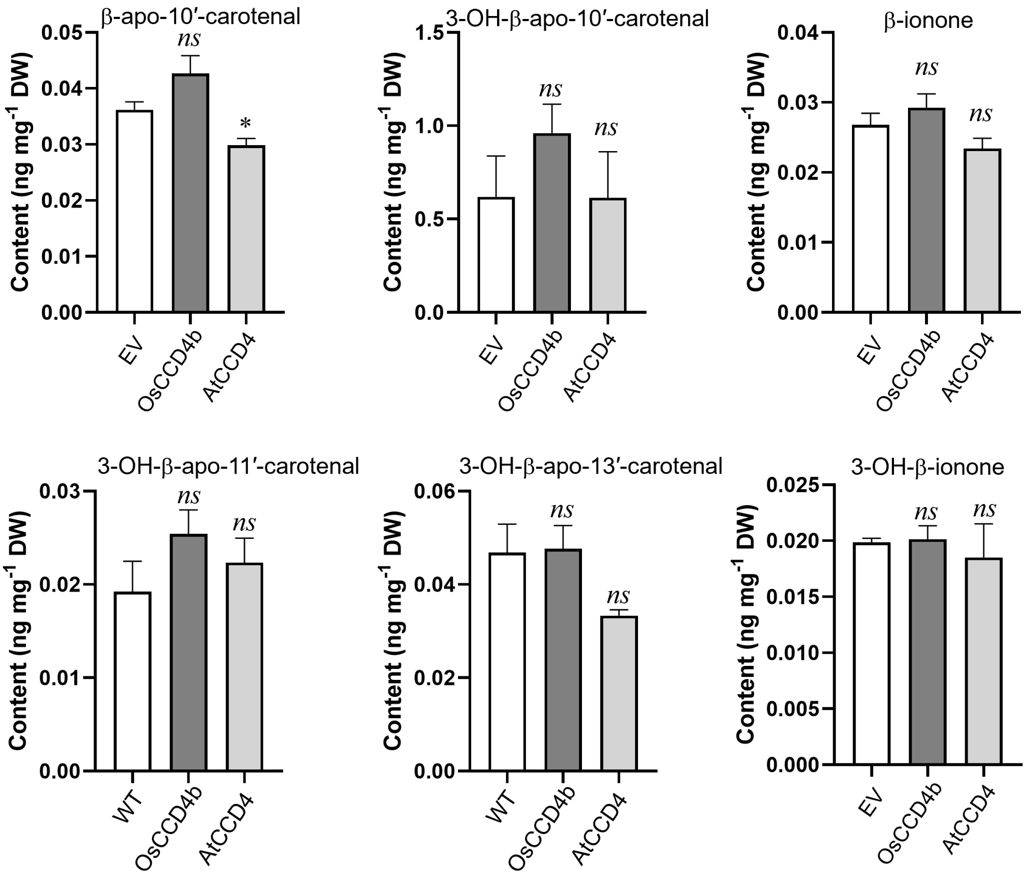

Figure S3. Apocarotenoid profiling of *Nicotiana* leaves transiently overexpressing *OsCCD4b* or *AtCCD4*. Identification and quantification of apocarotenoids in agroinfiltrated leaves were conducted by using UHPLC-HR-MS. Bars represent Standard Error of the Mean (SEM). Asterisks indicate statistically significant differences by Student's *t*-test: *P value < 0.05. "*ns*" indicates no significant difference.

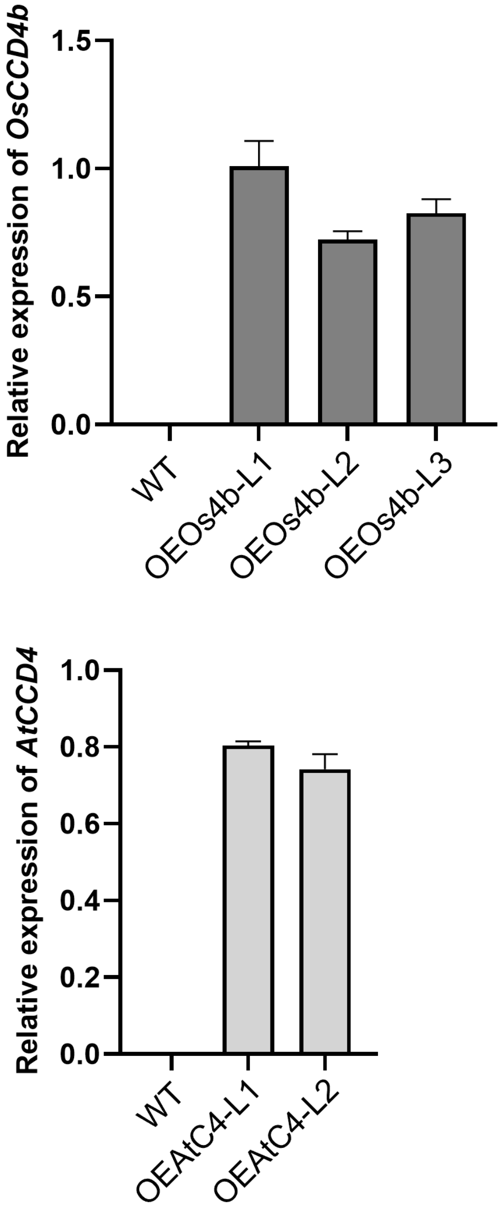

Figure S4. Relative expression levels of transgenes in independent *OsCCD4b-* or *AtCCD4-* overexpression citrus callus lines. OEOs4b and OEAtC4 represent different transgenic citrus callus lines overexpressing *OsCCD4b* and *AtCCD4*, respectively. Bars represent Standard Error of the Mean (SEM).

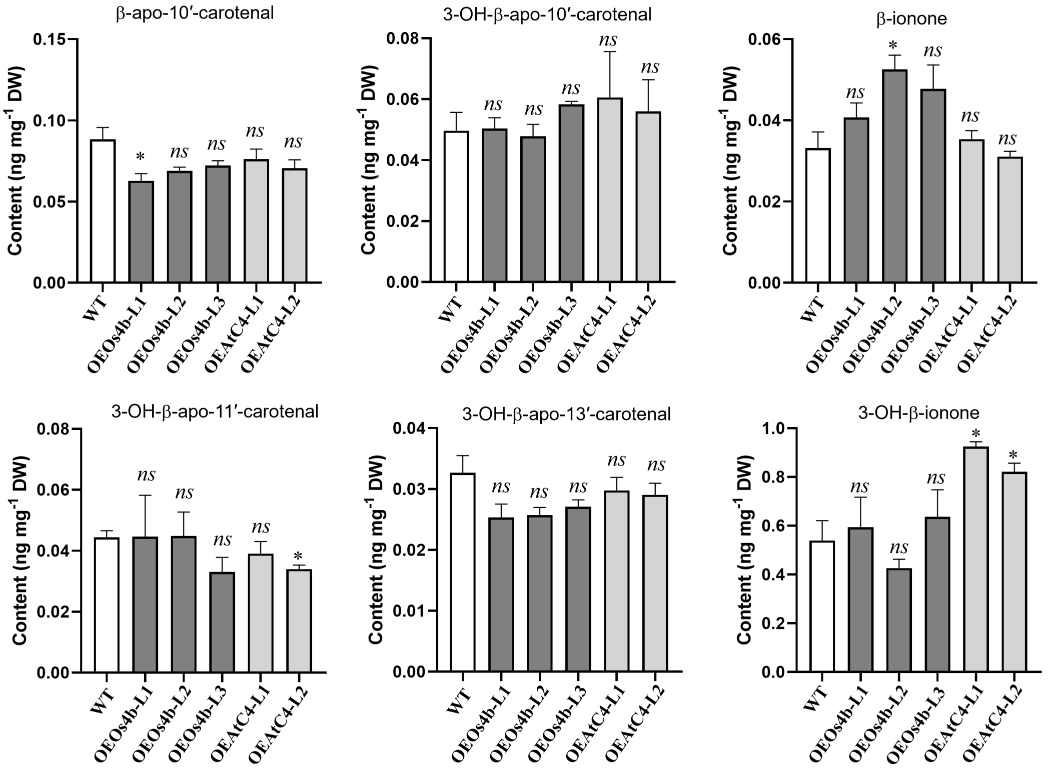

Figure S5. Apocarotenoid profiling in transgenic citrus callus overexpressing *OsCCD4b* or *AtCCD4*. OEOs4b and OEAtC4 represent different transgenic citrus callus lines overexpressing *OsCCD4b* and *AtCCD4*, respectively. Identification and quantification of apocarotenoids in transgenic citrus lines were conducted by using UHPLC-HR-MS. Bars represent Standard Error of the Mean (SEM). Asterisks indicate statistically significant differences by Student's *t*-test: **P* value < 0.05. "*ns*" indicates no significant difference.

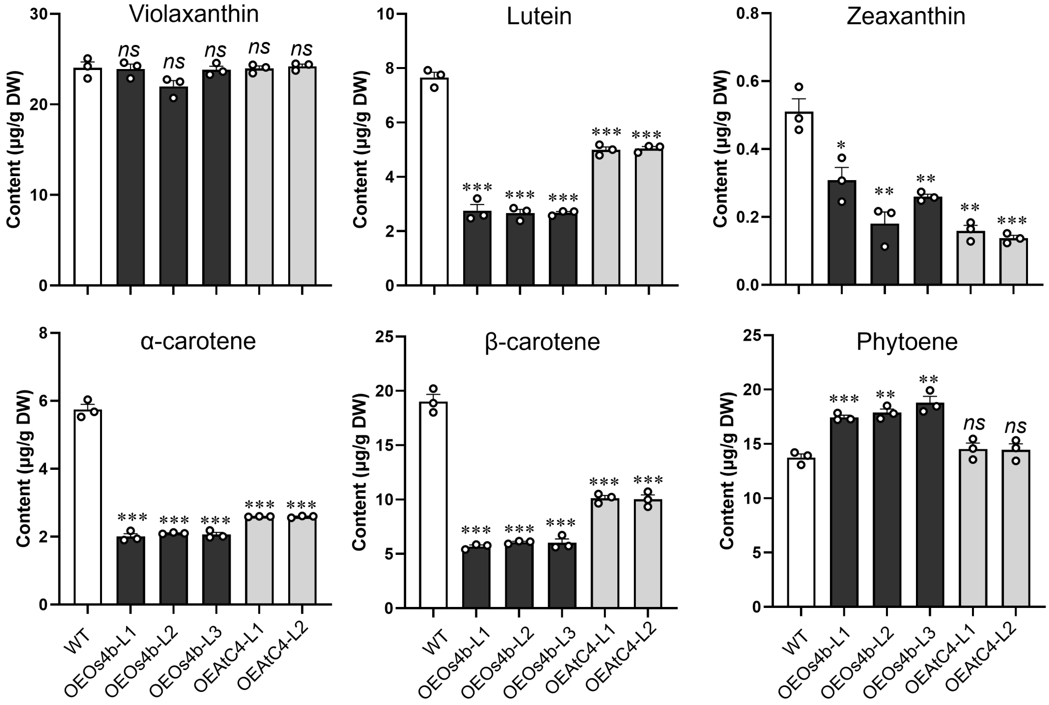

Figure S6. Carotenoid analysis of wild-type and *OsCCD4b* or *AtCCD4* overexpressing citrus callus lines. OEOs4b and OEAtC4 represent different transgenic lines overexpressing *OsCCD4b* and *AtCCD4*, respectively. The carotenoid analysis was performed by UHPLC-DAD. Bars represent Standard Error of the Mean (SEM). Asterisks indicate statistically significant differences by Student's *t*-test: **P* value < 0.05, ***P* < 0.01, and ****P* < 0.001, respectively. "*ns*" indicates no significant difference.

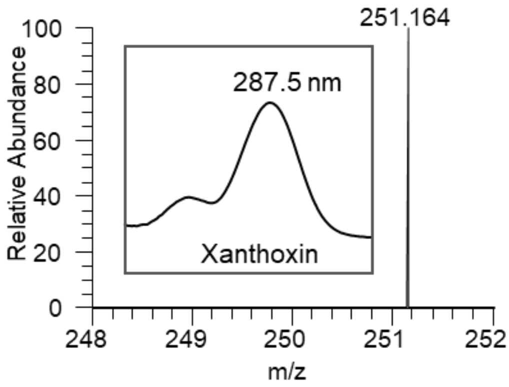

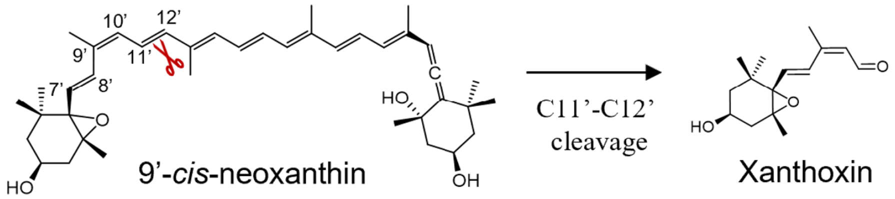

Figure S7. High-resolution MS and UV/vis spectra of xanthoxin.

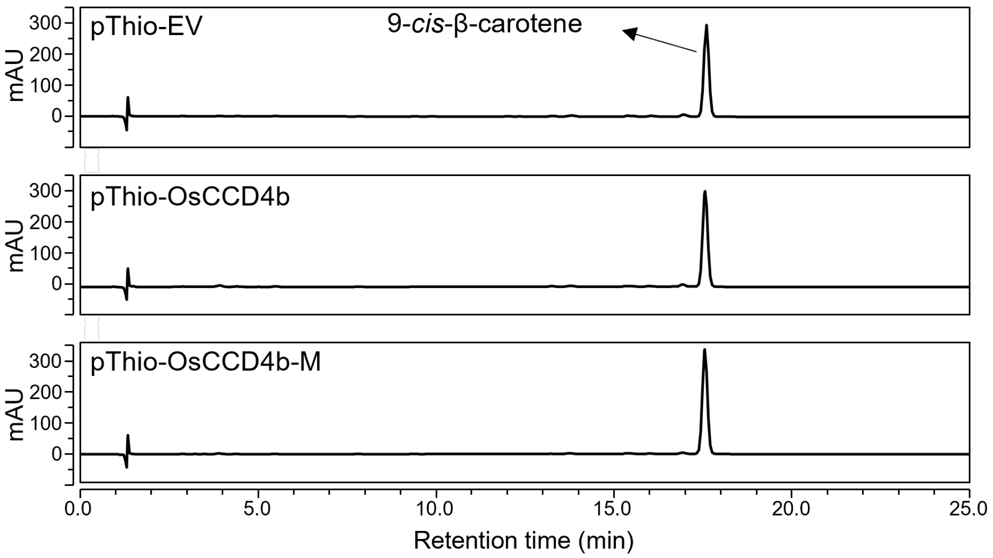

Figure S8. UHPLC analysis of the *in vitro* assays of OsCCD4b and its mutant using 9-*cis*-β-carotene as the substrate. No cleavage products were detected by UHPLC.

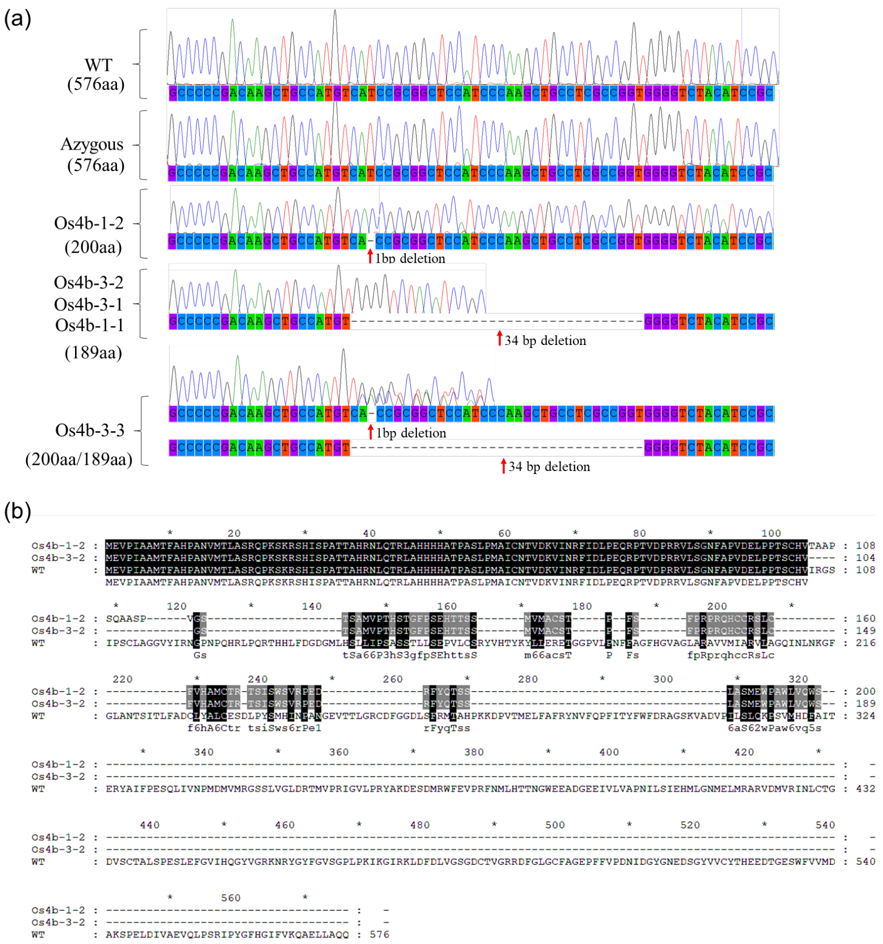

Figure S9. Mutations in *OsCCD4b* CRISPR knockout Nipponbare plants result in truncated proteins with loss of enzymatic activity. (a) Nucleotide sequence mutations in *OsCCD4b* from different CRISPR knockout mutants; (b) the corresponding translated protein sequences showing that mutations in the *OsCCD4b* coding region result in truncated proteins.

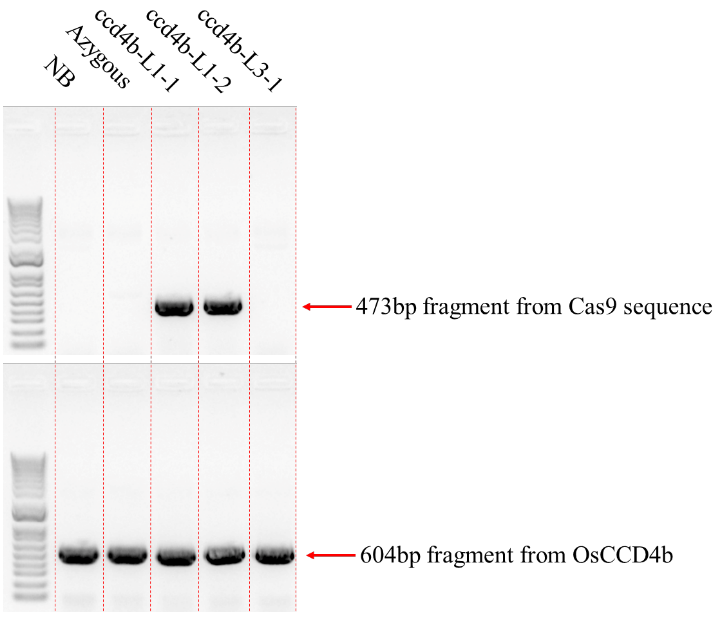

Figure S10. Identification of Cas9-free *OsCCD4b* CRISPR knockout mutant plants. The *ccd4b-L3-1* line was identified as a Cas9-free mutant.

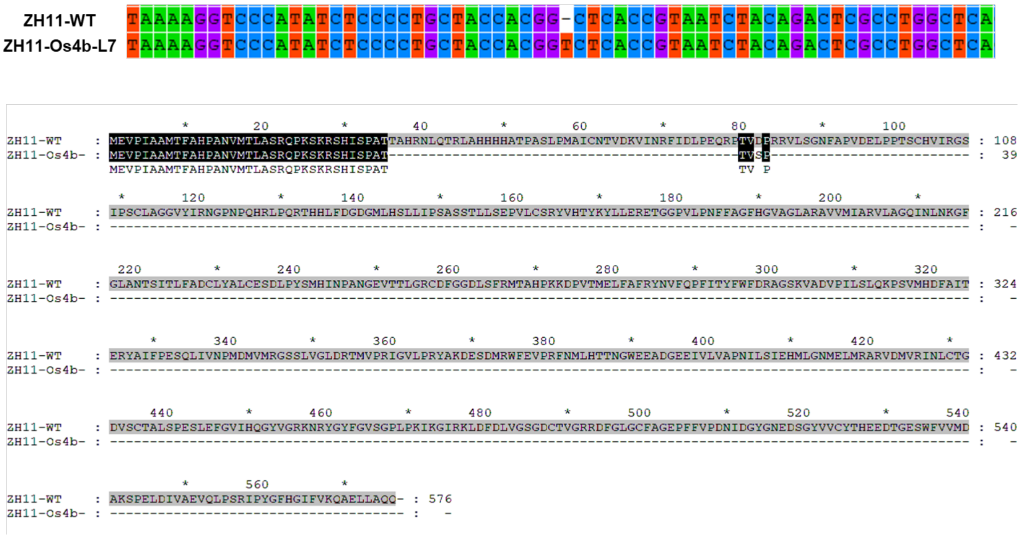

Figure S11. Sequencing analysis confirmed a *OsCCD4b* knockout CRISPR line in the Zhonghua 11 (ZH11) background.

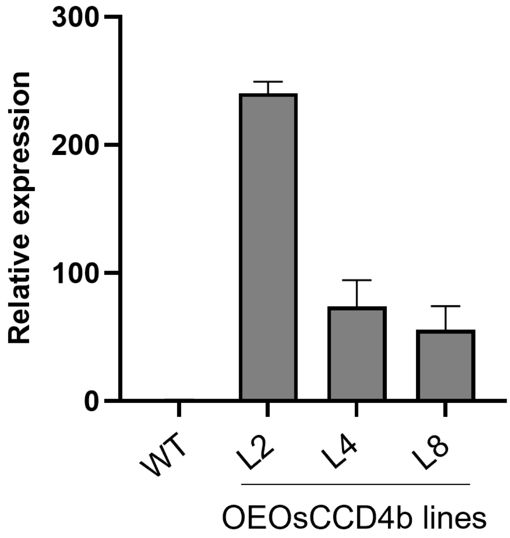

Figure S12. qRT-PCR confirmation of transgene overexpression in *OsCCD4b*-overexpressing Nipponbare plants. Bars represent Standard Error of the Mean (SEM).

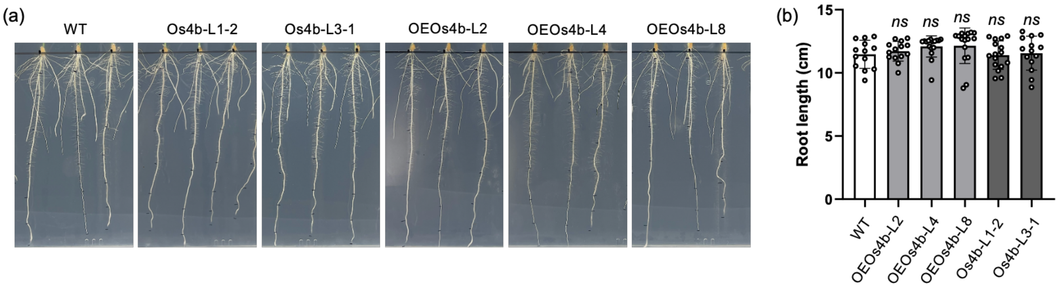

Figure S13. Root phenotypes of wild-type, *OsCCD4b* CRISPR knock-out, and overexpression line seedlings grown on agar medium. (a) Representative image of two-week-old rice seedlings grown on 0.8% agar-supplemented Hoagland medium. (b) Quantification of primary root length is shown in (a). Bars represent Standard Error of the Mean (SEM). Student's *t*-test, *ns*: not significant.

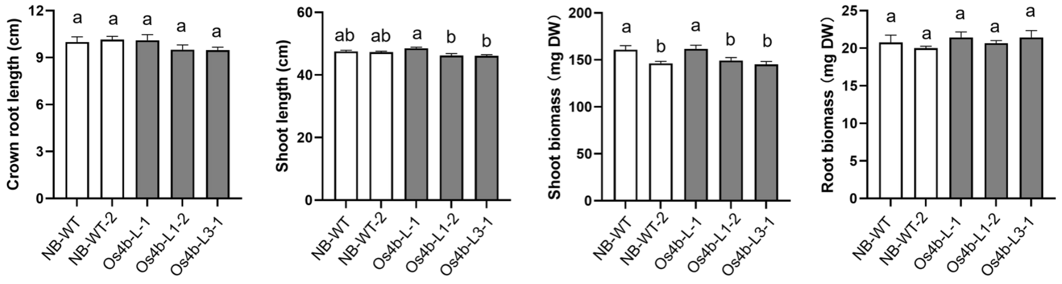

Figure S14. Phenotypic characterization of hydroponically grown Nipponbare wild-type and *OsCCD4b* CRISPR knockout mutant seedlings. Bars represent Standard Error of the Mean (SEM). The letters represent significantly different groups following ANOVA.DW represent dry weight.

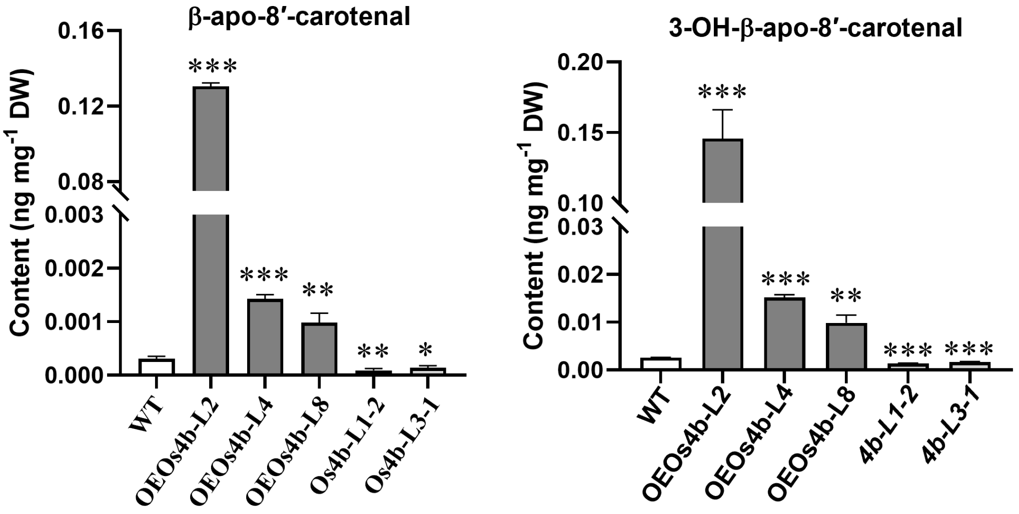

Figure S15. Quantification of β-apo-8′-carotenal and 3-OH-β-apo-8′-carotenal in different *OsCCD4b* overexpression lines and CRISPR knockout mutants. Bars represent Standard Error of the Mean (SEM). Asterisks indicate statistically significant differences by Student's *t*-test: *P value < 0.05, **P < 0.01, and ***P < 0.001, respectively.

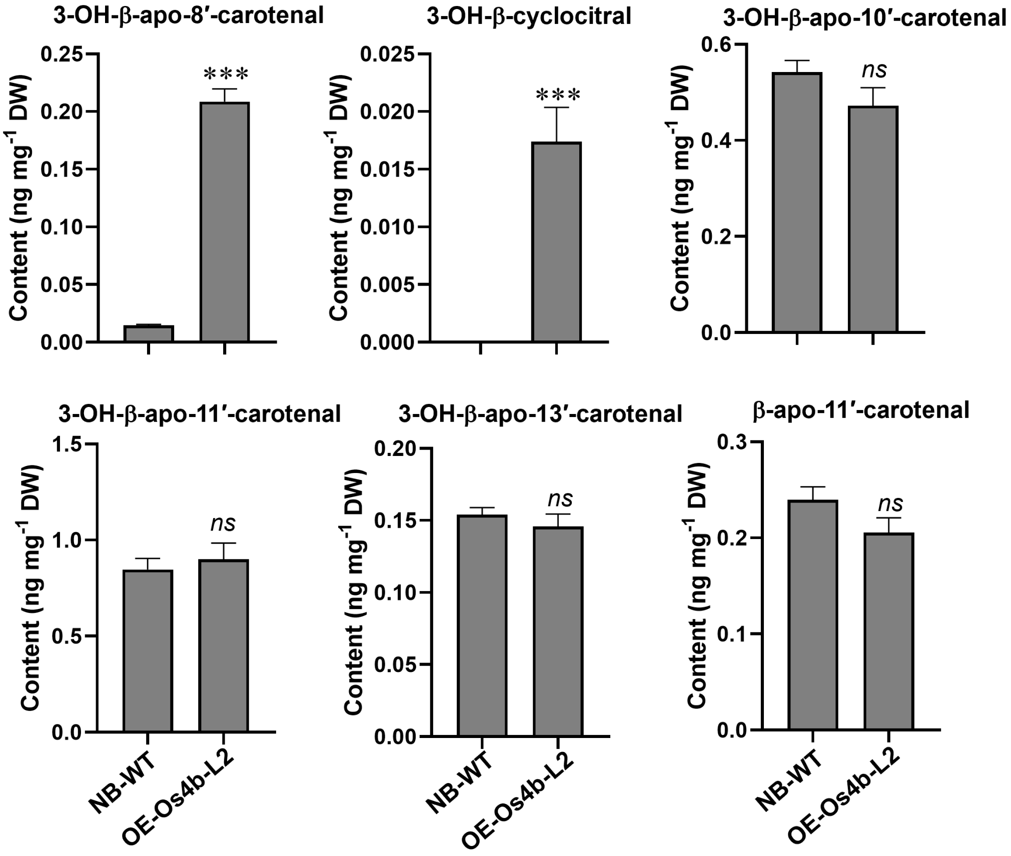

Figure S16. Apocarotenoid profiling in hydroponically grown shoots of Nipponbare wild-type and *OsCCD4b* overexpression lines. Identification and quantification of apocarotenoids were performed by using UHPLC-HR-MS. Bars represent Standard Error of the Mean (SEM). Asterisks indicate statistically significant differences by Student's *t*-test: ***$P < 0.001$. "*ns*" indicates no significant difference.

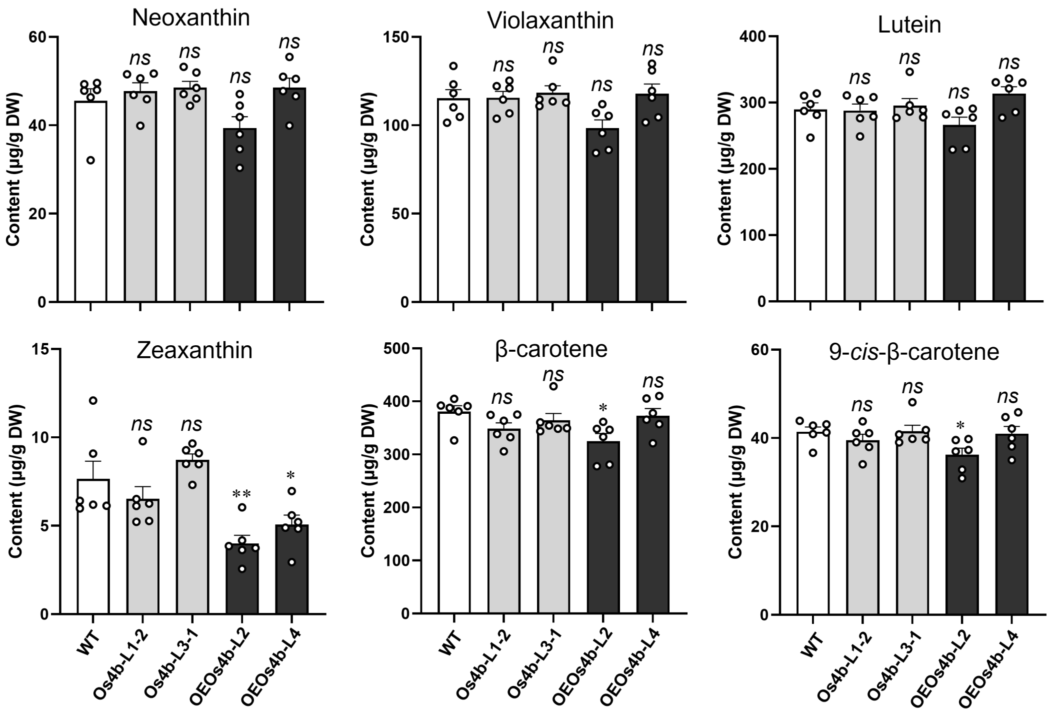

Figure S17. Carotenoid analysis in hydroponically grown shoots of wild-type, *OsCCD4b* overexpression lines, and CRISPR knockout mutants. *Os4b* and OEOs4b represent the *OsCCD4b* CRISPR knockout mutant and overexpression transgenic line, respectively. The carotenoid analysis was performed by UHPLC-DAD. Bars represent Standard Error of the Mean (SEM). Asterisks indicate statistically significant differences by Student's *t*-test: **P* value < 0.05 and ***P* < 0.01, respectively. "*ns*" indicates no significant difference.

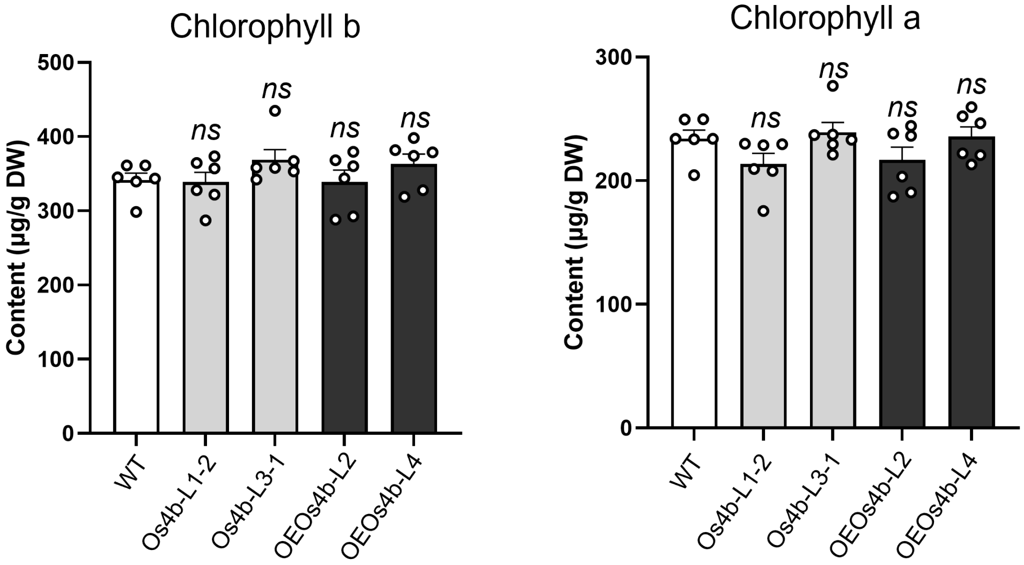

Figure S18. Chlorophyll levels in hydroponically grown shoots of wild-type, *OsCCD4b* overexpression lines, and CRISPR knockout mutants. *Os4b* and OEOs4b represent the *OsCCD4b* knockout mutant and overexpression transgenic line, respectively. The chlorophyll analysis was performed by UHPLC-DAD. Bars represent Standard Error of the Mean (SEM). "*ns*" indicates no significant difference.

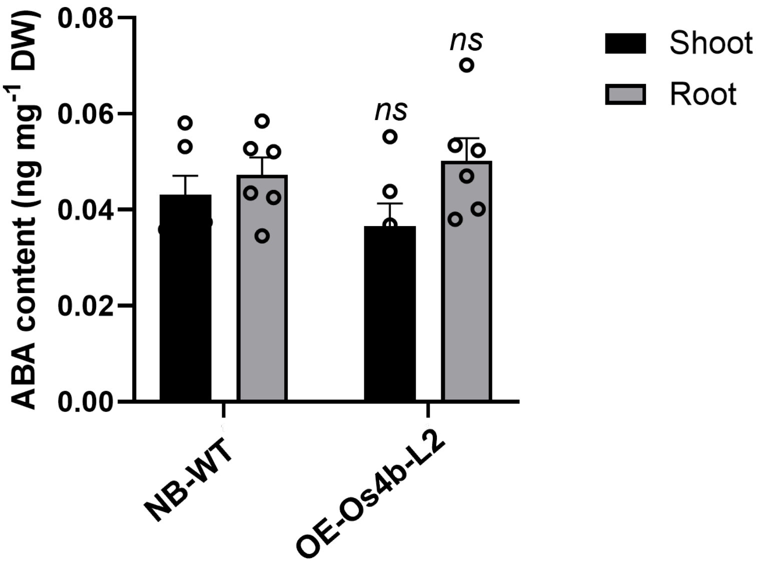

Figure S19. ABA quantification in shoots and roots of Nipponbare wild-type and *OsCCD4b* overexpression lines. ABA profiling was performed using UHPLC-HR-MS. Bars represent Standard Error of the Mean (SEM). "*ns*" indicates no significant difference.

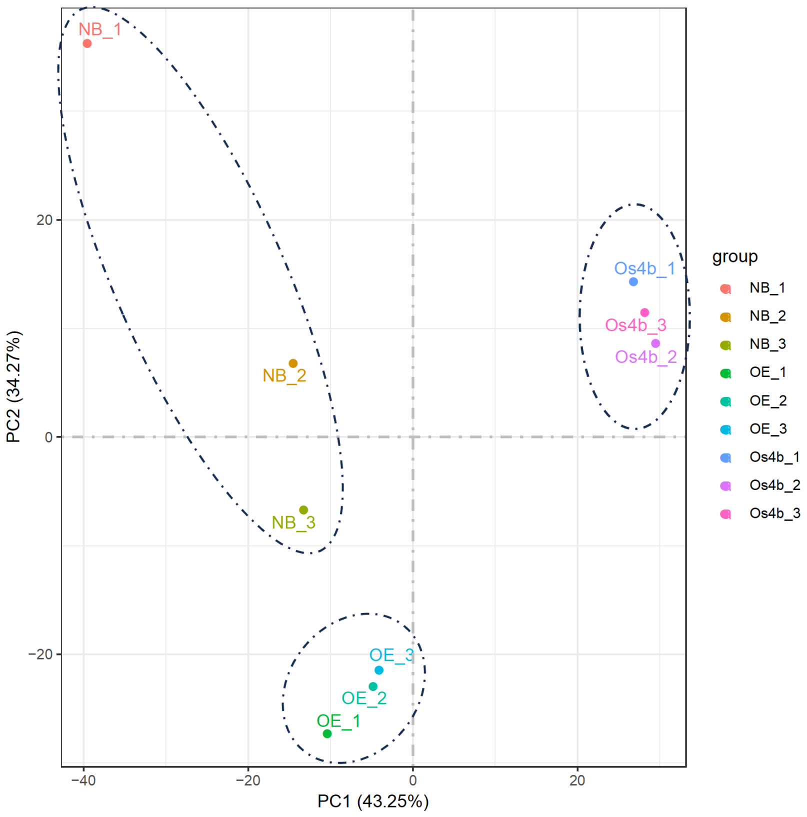

Figure S20. The principal component analysis (PCA) based on FPKM value of wild type (NB), *OsCCD4b* CRISPR knockout mutant, and overexpression lines obtained from RNAseq.

Supplementary Table:

Table S1. Sequences of primers used in this study.

Primer ID*	Primer sequences (5′→3′)
OsCCD4a-Thio-F	Forward: GGCGAATTCATGGCCAAGACCAAGACTACG
	Reverse: GACTCTAGATCATTGGTGCTGTGACTGGA
OsCCD4b-Thio-F	Forward: GGCGAATTCATGACTTTTGCCCACCCAGCC
	Reverse: GACTCTAGATTATTGTTGTGCGAGAAG
attB	Forward: GGGGACAAGTTTGTACAAAAAAGCAGGCT
	Reverse: GGGGACCACTTTGTACAAGAAAGCTGGGT
OsCCD4b-attB	Forward: AAAAAGCAGGCTTCATGGAGGTACCCATTGCT
	Reverse: AGAAAGCTGGGTGTTATTGTTGTGCGAGAAG
OsCCD4b-RT	Forward: CCAAACTTCTTCGCTGGCTT
	Reverse: GTGCATGGAGTAGGGAAGGT
AtCCD4-RT	Forward: GGTTCTCCGGTTGGTACTGA
	Reverse: GCATGAACCAGATCCATCCT
CitActin-RT	Forward: CCAAGCAGCATGAAGATCAA
	Reverse: ATCTGCTGGAAGGTGCTGAG

Note: * 'OsCCD4b-attB' primers were designed for overexpression vector construction using the BP recombination kit (Invitrogen); attB refers to the universal adaptor primers. 'RT' primers were used for qRT-PCR. 'Cit' indicates *Citrus*, 'At' indicates *Arabidopsis*, and 'Os' indicates rice.
